# Machine Learning-Assisted Hartree–Fock Approach for Energy Level Calculations in the Neutral Ytterbium Atom

**DOI:** 10.3390/e26110962

**Published:** 2024-11-08

**Authors:** Kaichen Ma, Chen Yang, Junyao Zhang, Yunfei Li, Gang Jiang, Junjie Chai

**Affiliations:** 1 National Key Laboratory of Particle Transport and Separation Technology, Tianjin 300180, China; makaichen@alu.scu.edu.cn (K.M.); junyao-z18@tsinghua.org.cn (J.Z.); liyunfei.2008@tsinghua.org.cn (Y.L.); 2 Research Institute of Physical and Chemical Engineering of Nuclear Industry, Tianjin 300180, China; 3 Institute of Atomic and Molecular Physics, Sichuan University, Chengdu 610065, China; gjiang@scu.edu.cn; 4 Key Laboratory of High Energy Density Physics and Technology, Ministry of Education, Chengdu 610065, China

**Keywords:** atomic calculation, machine learning, energy levels, entropy weight method, ytterbium

## Abstract

Data-driven machine learning approaches with precise predictive capabilities are proposed to address the long-standing challenges in the calculation of complex many-electron atomic systems, including high computational costs and limited accuracy. In this work, we develop a general workflow for machine learning-assisted atomic structure calculations based on the Cowan code’s Hartree–Fock with relativistic corrections (HFR) theory. The workflow incorporates enhanced ElasticNet and XGBoost algorithms, refined using entropy weight methodology to optimize performance. This semi-empirical framework is applied to calculate and analyze the excited state energy levels of the 4*f* closed-shell Yb I atom, providing insights into the applicability of different algorithms under various conditions. The reliability and advantages of this innovative approach are demonstrated through comprehensive comparisons with ab initio calculations, experimental data, and other theoretical results.

## 1. Introduction

Since the mid-20th century, driven by advancements in research methodologies and computational capabilities, atomic calculations have evolved from fundamental theoretical research into an increasingly popular and powerful tool spanning multiple fields, including plasma physics, astrophysics, spectroscopy, materials science, and molecular biology. The significance is not only manifested in its crucial role in interpreting spectral data, plasma diagnostics [1], and the study of atomic clocks [2], but also underscores that atomic calculations form the foundation for exploring the interactions of matter with various light fields and particles. In contrast, the currently known and available atomic structure data are relatively limited. The primary atomic structure databases are confined to a few key resources, such as the National Institute of Standards and Technology (NIST) Atomic Spectra Database [3], and the CHIANTI atomic database [4].

The expansion of atomic data is highly dependent on either advancement in atomic theory or our ability and methods to accurately predict the data. The Hartree–Fock (HF) method serves as the starting point for the majority of atomic and molecular electronic structure calculations. A series of post-Hartree–Fock methods, including multi-configuration Hartree–Fock (MCHF) [5] and multi-configuration Dirac–Hartree–Fock (MCDHF) [6], etc., have been developed to address the challenges in complex many-electron atomic systems, particularly the electron correlation effects and relativistic effects [7]. Corresponding to these theoretical advancements, several atomic calculation programs have emerged over the past few decades, including the Cowan code [8], developed in 1968 by R. D. Cowan based on the HFR method; CIV3 [9], proposed by A. Hibbert in 1975, based on configuration interaction; ATSP [10], developed by C. F. Fischer et al. in 1996, based on the MCHF method; GRASP [11,12], based on the MCDHF method, first proposed in 1989 and subsequently improved many times; RATIP [13,14], a relativistic calculation method developed based on GRASP; and FAC [15], a relativistic configuration interaction method that appeared in 2003.

Ab initio calculations present the optimal choice for calculating atomic structure data with minimal experimental input. However, these calculations are associated with high computational costs. Using a limited set of experimental values to guide machine learning algorithms in optimizing Cowan code’s calculations offers an alternative approach that maintains computational efficiency while improving accuracy, compared to implementing more sophisticated ab initio methods that require substantial computational resources and extremely complex atomic structure programs. Machine learning methodologies demonstrate particular efficacy for this task, owing to their capacity to be trained on sparse datasets, and be iteratively refined through the judicious incorporation of experimental values into the training set. Notably, they possess the potential to capture intrinsic correlation effects in complex atomic systems, particularly in addressing electron correlation effects and relativistic corrections where traditional approaches face computational challenges, thereby complementing existing theoretical frameworks. Various machine learning approaches have demonstrated breakthrough applications in multiple computationally intensive and accuracy-demanding fields of physics, such as characterizing physical models [16], creating interatomic potentials [17,18], and solving the Schrödinger equation [19,20].

A serious problem in machine learning-assisted atomic structure calculations is the selection of an appropriate machine learning algorithm. There is no established method for determining the best-suited algorithm for a specific problem a priori, necessitating repeated testing. Thus, a robust and scalable framework is essential.

In this study, we chose the Cowan code, based on HFR theory, as our ab initio computational platform. The Cowan code inherently incorporates least-squares fitting (LSF), resulting in the data structures generated during its computational process being highly conducive to regression-based machine learning algorithms. Furthermore, its relatively streamlined program architecture and robust performance in calculating the spectra of transition elements, including lanthanides and actinides, facilitates the implementation of flexible data interfaces [21,22,23]. Based on this, we developed a machine learning-assisted atomic structure calculation workflow with four improved machine learning algorithms embedded.

The energy level structure of ytterbium (Yb) is characterized by distinct features, including large inter-configuration energy gaps and densely populated energy level clusters. Coupled with the relative abundance of experimental data, these properties render Yb an ideal system for investigating the atomic structure of lanthanide elements.

This article is organized as follows: In Section 2, we detail the machine learning-assisted atomic structure calculation workflow, along with the four newly designed machine learning algorithms. In Section 3, we apply this workflow to compute selected excited state energy levels of Yb I, followed by a comparative analysis to identify the appropriate application scenarios for each algorithm. Finally, Section 4 concludes the work.

## 2. Calculation Methods

**Figure 1:**
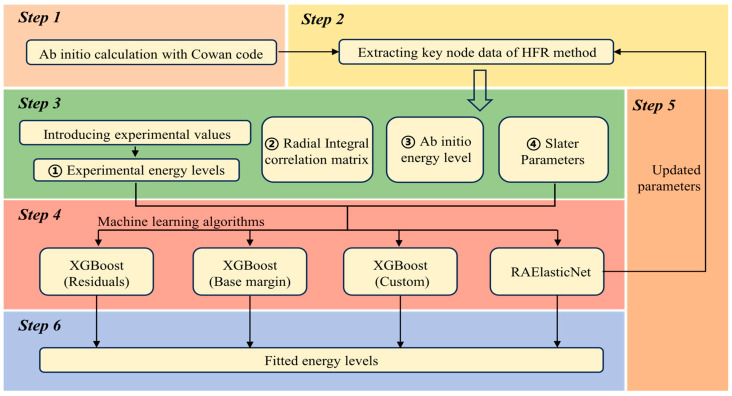
Step-by-step diagram of the machine learning-assisted atomic structure calculation workflow. Step 1: Initial calculation; Step 2: data extraction; Step 3: data preparation; Step 4: machine learning fitting calculations; Step 5: parameter refinement; Step 6: results evaluation.

This study proposes a novel machine learning-assisted workflow for atomic structure calculations. We develop a versatile and scalable framework capable of both multi-atomic system calculations and flexible integration of diverse machine learning algorithms, tailored to specific research requirements. Figure 1 presents the steps and components of the workflow. We elaborate on each step of the workflow, demonstrating how traditional computational methods collaboratively operate with machine learning algorithms, as well as how newly designed algorithms can be seamlessly integrated into the framework.

### 2.1. Machine Learning-Assisted Atomic Structure Calculation Workflow

**Step 1: Initial calculation.** Ab initio calculations based on the HFR theory are accomplished by Cowan code.

In the HFR theory, ab initio single-electron radial wave functions R(r) for all the subshells of the considered configurations will be computed first, and as a result some important energy parameters, called Slater parameters, can be obtained for each configuration, such as center-of-gravity configuration energies $E_{av}$, Slater direct integral $F^{k}$, Slater exchange integral $G^{k}$, radial integral related to the spin–orbit interaction $\zeta_{j}$, which in terms of the radial portion of a single-electron wave function R(r) can be defined as:

(1) $$F^{k}\left(l_{i},l_{j}\right)=\int_{0}^{\infty }\int_{0}^{\infty }\frac{{2r}_{<}^{k}}{r_{>}^{k+1}}\times R_{i}\left(r_{1}\right)R_{j}\left(r_{2}\right)R_{i}\left(r_{1}\right)R_{j}\left(r_{2}\right)r_{1}^{2}r_{2}^{2}dr_{1}dr_{2},$$

(2) $$G^{k}(l_{i},l_{j})=\int_{0}^{\infty }\int_{0}^{\infty }\frac{{2r}_{<}^{k}}{r_{>}^{k+1}}\times R_{i}(r_{1})R_{j}(r_{2})R_{i}(r_{2})R_{j}(r_{1})r_{1}^{2}r_{2}^{2}dr_{1}dr_{2},$$

and:

(3) $$\zeta_{j}=\int_{0}^{\infty }\frac{1}{r}\;\left(\frac{dV}{dr}\right){\left|R_{j}(r)\right|}^{2}dr.$$

These electrostatic and spin–orbit interaction parameters are crucial for energy level calculations and atomic Hamiltonian matrix element construction. According to Slater-Condon theory [24], utilized in the HFR method, these elements can be characterized as follows:

(4) $$\begin{array}{l} {\delta H}_{ab}=\delta_{ab}E_{av}+\sum_{j=1}^{q}\left[\sum_{k>0}{\left(f^{k}\left(l_{j},l_{j}\right)\right)}_{ab}F^{k}\left(l_{j},l_{j}\right)+{\left(d_{j}\right)}_{ab}\zeta_{j}\right]+\\ \sum_{i=1}^{q-1}\sum_{j=i+1}^{q}\left[\sum_{k>0}{\left(f^{k}\left(l_{i},l_{j}\right)\right)}_{ab}F^{k}\left(l_{i},l_{j}\right)+\sum_{k}{\left(g^{k}\left(l_{i},l_{j}\right)\right)}_{ab}G^{k}\left(l_{i},l_{j}\right)\right]\times (|l_{i}-\\ l_{j}|\leq k\leq l_{i}+l_{j}), \end{array}$$

where $f^{k}$, $g^{k}$, and $d_{j}$ are the angular coefficients of the Slater parameters $F^{k}$, $G^{k}$, and $\zeta_{j}$, respectively. Next, for each possible value of total angular momentum J, energy matrices are set up and diagonalized to attain eigenvalues and eigenvectors, respectively.

**Step 2: Data extraction.** We implemented customized modifications to the Cowan code. Based on HFR theory, we established data output interfaces and preliminary data formatting processes at key nodes of the program. This approach enables real-time capture and export of key parameters during the ab initio calculation process, including the aforementioned series of electrostatic and spin–orbit interaction Slater parameters: $E_{av}$, $F^{k}$, $G^{k}$, and $\zeta_{j}$, as well as the radial integral correlation matrix that connects these parameters to energy levels. This method ensures a direct correlation between the extracted data and the computational process, minimizing intermediate processing steps and thereby enhancing the accuracy and traceability of the data.
**Step 3: Data preparation.** This phase integrates multi-source data, including experimental energy levels from the NIST database, ab initio calculated energy levels, and the electrostatic and spin–orbit interaction Slater parameters along with their corresponding correlation matrices. In this step, we also perform comprehensive preprocessing on these datasets prior to machine learning training. This involves constructing a pipeline for data cleaning and standardization, thereby transforming the data into a correlation matrix encompassing both feature variables and target values, suitable for machine learning algorithms.
**Step 4: Machine learning fitting calculations.** The machine learning algorithm performs the fitting calculations in this step using the correlation matrix containing all the preprocessing information, where the radial integral correlation matrix is used to form the feature variables, and the NIST [3] experimental energy level values are used as the target values. We designed this step as a *drawer* to freely integrate different machine learning algorithms. To facilitate computation, we employ both linear (ElasticNet) and tree-based (XGBoost) models. These algorithms are tailored and optimized for the specific characteristics of the atomic structure data, as elaborated in Section 2.2.
**Step 5 and 6: Results evaluation and parameter refinement.** We evaluate the performance of the machine learning algorithms using the root mean square error, and the mean absolute error to measure the accuracy of fitting the computational energy level data. We use five-fold cross-validation and grid search to update the hyperparameters to optimize the generalization ability of different machine learning algorithms and finally attain the best computational results. Furthermore, our enhanced ElasticNet model refines Slater parameters, facilitating an iterative optimization process when reintroduced at Step 2.

### 2.2. Machine Learning Algorithms

Two categories of machine learning algorithms were employed: linear models and tree-based models, including Residuals Adaptive ElasticNet (RAEN) as a linear model, along with a suite of ensemble tree models comprising XGBoost-Residuals (XGB-R), XGBoost-Base margin (XGB-B), and XGBoost-Custom (XGB-C). Considering the intrinsic linear nature of the Slater parameter correlation matrix (feature matrix) derived from the Cowan code, which incorporates LSF as its own fitting calculation method, we additionally designed linear models for comparative analysis. Similar studies were conducted by using ridge regression models [25].

Ensemble tree models, renowned for their high predictive accuracy, have been established as reliable tools for addressing computational challenges in complex microscopic systems, such as in the field of materials science [26]. Given that ab initio calculations can provide initial predictions as target values—a rare advantage in machine learning tasks—the XGBoost model, with its high predictive accuracy, emerges as an ideal choice. This is particularly due to its key feature of residual learning capability, which focuses on learning the discrepancies between initial predictions and actual values. Consequently, we custom-designed XGB-R, XGB-B, and XGB-C to address various data characteristics and application scenarios.

#### 2.2.1. Linear Model

In the formulation of our model, we define the parameter vector β as follows:

(5) $$\beta_{0}={\left[E_{AV},G^{k},F^{k},\zeta_{j}\right]}^{T},$$

the general form of linear model can be expressed as:

(6) $$y={\left[1,\;x_{1},x_{2},\;\ldots ,\;x_{n}\right]}^{T}\beta .$$

The Residuals Adaptive ElasticNet (hereafter referred to as RAEN) employed in this study maintains the sparsity and regularization advantages of the standard ElasticNet [27] while incorporating two key enhancements: a dynamic sample weighting mechanism to balance the importance of different samples, and a residual penalty term to focus particularly on samples that are difficult to fit. This structural design underpins the nomenclature RAEN. In comparison to the loss function of the standard ElasticNet, which is expressed as:

(7) $$L\left(\beta \right)=\frac{1}{2n}\sum {\left(y_{i}-{x_{i}}^{T}\;\beta \right)}^{2}+\alpha \;[\rho {\left\|\beta \right\|}_{1}+\frac{1-\rho }{2}],$$

the loss function of RAEN is:

(8) $$L\left(\beta \right)=\frac{1}{2n}\sum \;{w_{i}\;\left(y_{i}-{x_{i}}^{T}\;\beta \right)}^{2}+\alpha \;[\rho {\left\|\beta \right\|}_{1}+\frac{1-\rho }{2}],$$

where $w_{i}$ is defined as:

(9) $$w_{i}=\frac{1}{M+P+R_{i}},$$

composed of the mean squared error (MSE) M, parameter control term P, and residual constraint term $R_{i}$:

(10) $$M=\frac{1}{n}\sum {\left(y_{i}-\hat{y_{i}}\;\right)}^{2},$$

(11) $$P=\frac{1}{p}\sum \left|\beta_{j}-\beta_{0}\right|,$$

(12) $$R_{i}=|y_{i}-\hat{y_{i}}\;|.$$

Here, ${x_{i}}^{T}\beta$ and $y_{i}$ are the fitting calculation (prediction) and experimental energy level, $\hat{y_{i}}$ is ab initio value, α and ρ are the regularization coefficients controlling the $L_{1}$ and $L_{2}$ penalty terms, respectively. In the computational process, we directly treat β as the adjustable model parameters (coefficients of the matrix), using $\beta_{0}$ as the initial point for updates. The optimal energy values and parameter vectors are obtained through an iterative process that minimizes the loss function.

#### 2.2.2. Tree-Based Model

Tree-based models, particularly ensemble tree methods, do not possess a concept analogous to the *coefficients* found in linear models. Instead, they rely on the aggregation of multiple tree learners for computation, a process known as *boosting*.

Tree-based models perform calculations through recursive node partitioning, which directly yields energy level predictions but does not generate *coefficients* like those in linear models. Consequently, these models do not require Slater parameters. They perform calculations solely based on initial predictions (ab initio) and the radial integral correlation matrix. The final prediction of the model is the cumulative sum of the predictions from multiple tree models. The standard XGBoost algorithm [28], an efficient and robust gradient boosting decision tree algorithm, can be represented as:

(13) $$y_{i}=\varphi (x_{i})=\sum_{k=1}^{M}f_{k}\;(x_{i}).$$

The loss function is:

(14) $$L\left(\varphi \right)=\sum_{i=1}^{N}\;l\left(y_{i},\hat{y_{i}}\right)+\sum_{k=1}^{M}\;\Omega \left(f_{j}\right),$$

Here, *l* is a differentiable convex loss function which measures the difference between the prediction $\hat{y_{i}}$ and the target $y_{i}$. The term Ω penalizes the complexity of the model.

In this study, we propose a modified version of the standard XGBoost model, termed XGBoost-Residuals (XGB-R). This variant alters the prediction direction of the model as follows:

(15) $$\varphi \left(x_{i}\right)=y_{i_{0}}-y_{i},$$

where $y_{i_{0}}$ is the initial prediction. The model’s fitting objective is transformed from the final values to the residuals of previous iteration. The final prediction is obtained through the cumulative summation of the results from each fitting iteration. By directly learning the residuals, the prediction accuracy is significantly enhanced. 

XGBoost-Base margin (XGB-B) incorporates the initial prediction (ab initio) into the base learner, denoted as $f_{j}(x_{i},y_{i_{0}})$. This approach uses the initial prediction as a baseline, with the model’s fitting results representing adjustments to this baseline.

XGBoost-Custom (XGB-C) modifies the objective function, defined by the equation:

(16) $$L_{Custom}\left(\varphi \right)=\sum_{i=1}^{N}\left[\;w_{1}l(y_{i},\hat{y_{i}}\right)+w_{2}\lambda ({y_{i_{0}}-y_{i})}^{2}]+\sum_{k=1}^{M}\;\Omega (f_{j}),$$

where $l(y_{i},\hat{y_{i}})$ is the prediction loss, $\lambda ({y_{i_{0}}-y_{i})}^{2}$ is the residual constraint term, and $w_{1}$, $w_{2}$ are entropy-based weights determined by:

(17) $$w_{j}=\left(1-H_{j}\right)/\sum_{k=1}^{2}\left(1-H_{k}\right),\;j=1,\;2,$$

(18) $$H_{j}=-\sum_{i=1}^{N}p_{ji}\times \ln\left(p_{ji}\right),\;j=1,\;2.$$

Here, $H_{j}$ represents the information entropy [29] of the j-th term (j=1 for prediction loss, j=2 for residual constraint), $p_{ji}$ are the normalized values of the prediction loss and residual constraint terms, respectively.

By incorporating both the custom objective function and evaluation metrics, XGB-C aims to achieve a more optimal balance between residual constraints and the influence of initial predictions. The introduction of the entropy weight method enhances the model’s adaptability, enabling dynamic adjustment of the relative importance of prediction accuracy and consistency with initial predictions based on data distribution.

## 3. Results and Discussion

To elucidate the distinctive characteristics of four computational methods—RAEN, XGB-R, XGB-B, and XGB-C—in calculating complex energy level structures, we selected the Yb I atom for analysis. This element was chosen due to its large inter-configuration energy gaps and densely populated energy level clusters.

We calculated the energy levels of multiple excited states of Yb I, categorized by odd and even parity, relative to the ground state configuration [Xe]4*f*^14^6*s*^2^. The calculated relative energies are systematically compared with the experimental data provided by the NIST Atomic Spectra Database [3].

### 3.1. Calculations of Yb I Even-Parity Excited States

We selected multiple outer-shell excited states, specifically those maintaining an invariant 4*f*^14^ closed-shell core and involving only *s*, *p*, and *d* orbitals. This enabled us to analyze the characteristics of different computational methods when fitting large datasets of energy levels.

Utilizing the Cowan code, we conduct ab initio calculations of Yb I even-parity excited state energy levels based on HFR theory. We then integrated high-precision experimental energy levels to perform fitting calculations using various regression methods. The results of all possible terms are listed in Table 1.

The ab initio calculation results are compared with the experimental values provided by NIST to compute ∆E (the absolute errors) and R (the root mean square error, RMSE, $R=\sqrt{\sum_{i}^{N}\frac{{\left(y_{i}-\hat{y_{i}}\right)}^{2}\;}{N}}$, where $y_{i}$ and $\hat{y_{i}}$ are the fitting calculation and experimental energy level from NIST, and N is the number of energy levels). These metrics are used to evaluate the accuracy of the new computational methods.

We report ∆E for each term and R for all energy levels incorporated in our calculations. A lower R-value signifies higher computational accuracy, with this metric being particularly sensitive to extreme deviations and outliers. Consequently, regression model predictions that substantially diverge from experimental values are deemed unacceptable. Such discrepancies often indicate either overfitting of the machine learning model or, more critically, a failure to capture the underlying patterns in the Slater parameter features, rendering the model incapable of accurate predictions.

**Figure 2:**
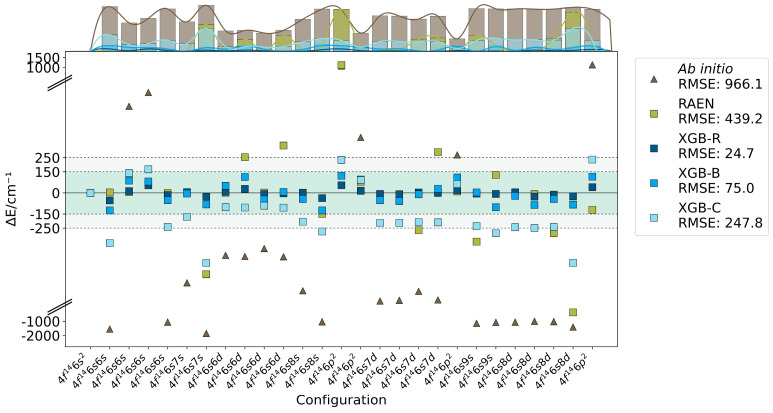
Errors of even-parity excited state energy levels for Yb I calculated using ab initio method, RAEN, XGB-R, XGB-B, and XGB-C. Top marginal histograms show error distributions for each method. The vertical axis is broken (indicated by //) to accommodate the large range of errors.

It must be acknowledged that the ab initio calculations using the Cowan code have largely elucidated the energy level positions and structures, albeit with limited accuracy (R-value is 966.1). This, however, represents the limit of the HFR method’s capabilities. The reduction in R (R-value is 439.2, 24.7, 75.0, and 247.8) demonstrates that the four novel methods, through the incorporation of select high-purity experimental energy levels for fitting, have substantially enhanced computational precision. Figure 2 provides a more intuitive illustration of this improvement from the perspective of error.

Ab initio calculations (denoted by triangles) exhibit substantially larger deviations compared to the other four computational methods (represented by squares). This pronounced disparity is further evidenced in the marginal histograms, where the ab initio method displays a distinctly prominent bar. Our RAEN model also demonstrates deviations, notably in computing the 4*f*^14^6*p*^2^ configuration, where its performance is surpassed even by ab initio calculations.

This phenomenon is intrinsically linked to the substantial energy level spacing between distinct spectral terms of the 6*p*^2^ configuration, with the ³P state demonstrating pronounced exchange interaction among parallel spin electrons. Moreover, single linear models often exhibit diminished efficacy when concurrently processing extensive datasets. A comprehensive analysis of linear model behavior on reduced sample sizes is presented in Section 3.2.

Tree-based methods demonstrated excellent performance with this dataset. These XGBoost models, trained on experimental energy levels, accurately capture the relationship between Slater parameter features and energy level values in the HFR method. This numerical approach augments the HFR theory by incorporating certain electron-electron interactions previously neglected in the original formulation. 

The lower R-values serve as a clear validation of this approach. Moreover, when processing large-interval energy level sequences across different energy clusters, the computational results of all three XGBoost models demonstrate stability. This was particularly evident in the XGB-R and XGB-B models, where the maximum absolute error is constrained within 150 cm^−1^, and both models achieve R-values below 100 cm^−1^.

The XGB-C model demonstrates mediocre accuracy for this dataset, an outcome anticipated in the initial design phase. This underscores an inherent challenge in the fitting process: overfitting. As the number of experimental energy levels utilized for training expands, the overfitting phenomenon becomes more pronounced in complex tree-based models.

**Figure 3:**
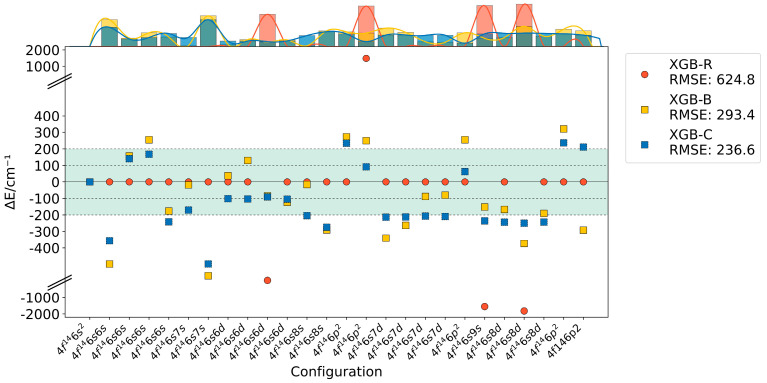
Errors in even-parity excited state energy levels of Yb I calculated by XGB-R, XGB-B, and XGB-C models with an expanded training set. Top marginal histograms show error distributions for each method. The vertical axis is broken (indicated by //) to accommodate the large range of errors.

Upon augmenting the volume of experimental energy levels in the training set, the XGB-R model’s calculations exhibit an ostensibly “*perfect*” fit to the majority of experimental values, as illustrated in Figure 3. However, this apparent precision belies severe overfitting, evidenced by a R of 624.8. The R-value exhibits a substantial increase, accompanied by significant deviations from experimental values for several energy levels: ^3^D_3_ of 4*f*^14^6*s*6*d*, ^3^P_1_ of 4*f*^14^6*p*^2^, ^3^S_1_ of 4*f*^14^6*s*9*s*, and ^3^D_2_ of 4*f*^14^6*s*8*d*. Enhanced residual learning enables it to effectively capture experimental data characteristics. However, overly stringent residual constraints compromise generalization capability. The XGB-B model, utilizing ab initio calculations as its initial prediction baseline, encounters fitting disruptions when training energy levels significantly deviate from ab initio results. The XGB-C model establishes an equilibrium between the aforementioned approaches by employing the entropy weight method to dynamically and adaptively adjust the influence of ab initio calculations and residual constraints. Additionally, the incorporation of the entropy weight method in XGB-C enables dynamic adjustment based on data distribution, particularly the distribution of energy levels across neighboring configurations. This approach potentially leads to enhanced performance in capturing complex configuration interactions.

### 3.2. Small-Sample Analysis of Linear Models for Yb I Excited States

For linear models, we use Cowan fit and RAEN to calculate a set of even-parity excited state energy levels with a relatively small energy span. As the dataset size decreased, the advantages of linear models became increasingly apparent in Table 2. 

The Cowan code includes an optional fitting module that employs the LSF method to optimize calculations against experimental values. We denote the results from this approach as “Cowan fit”. Given that both LSF and RAEN are linear methods, we include this approach in our comparative analysis.

The R of the RAEN model (R-value of 114.1) showed a significant improvement compared to its performance on larger datasets, and was substantially better than the fitting results from the built-in calculation of the Cowan code (R-value of 1040.8).

**Figure 4:**
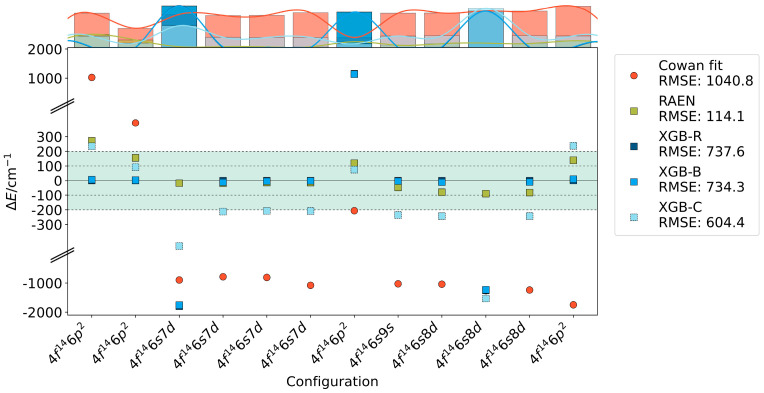
Errors in small-sample even-parity excited state energy levels of Yb I calculated using Cowan fit, RAEN, XGB-R, XGB-B, and XGB-C methods. Top marginal histograms show error distributions for each method. The vertical axis is broken (indicated by //) to accommodate the large range of errors.

Cowan fit utilizes the LSF method, which is susceptible to overfitting in high-dimensional or multicollinear datasets, even with small sample sizes. RAEN, constrained by combined $L_{1}$ and $L_{2}$ regularization terms, effectively mitigating this issue. Complex models such as XGB are similarly prone to overfitting with limited samples, as demonstrated in Figure 4.

Similar studies were conducted by using ridge regression models [25]. In their approach, experimental values were incorporated discretely for each configuration, effectively creating smaller sample subsets for the fitting process. This approach exemplifies the segmented fitting paradigm prevalent in linear models. We applied an analogous segmented fitting technique to the RAEN model when computing odd-parity excited state energy levels of Yb I. This method yielded a great enhancement in computational precision.

Another advantage of linear models is that they directly yield updated Slater parameters ($E_{av}$, $F^{k}$, $G^{k}$, and $\zeta_{j}$) after fitting calculation. In linear models, these parameters are treated as directly adjustable variables (feature coefficients). In contrast, within the context of HFR theoretical calculations, these parameters assume a critical role as fundamental components in constructing the Hamiltonian. The RAEN model updated partial parameters, as shown in Table 3, and through a feedback loop, it corrected the Hamiltonian calculations in the Cowan code.

Tree-based models perform calculations through node partitioning, thus they can only directly fit energy level values and do not incorporate the concept of these types of parameters. However, our analysis of XGB model calculation results indicates that as the number of built-in parameters in tree models increases and complexity rises, computational accuracy indeed improves.

Moreover, the XGB model employs a second-order Taylor expansion to approximate the loss function, so we can put forward an interesting but reasonable conjecture that for the HFR theory, if the Hamiltonian is expanded into more orthogonal radial integral terms, perhaps the computational accuracy of ab initio will also be improved, but of course, this requires more work to verify. 

Additionally, we attempted to calculate the energy level using the support vector regression (SVR) method. However, the results were unsatisfactory, suggesting that kernel methods may not be well-suited for processing these types of data. We also applied these methods to calculate odd-parity excited state energy levels for the Yb I in Table 4. The results showed improvement over the ab initio method. Notably, the XGB-R model achieved R-value of 67.2, with the relative error controlled within 0.5%.

Furthermore, it is important to note that in the atomic and molecular field, the predictability of theoretical models is intrinsically linked to their capability to provide specific data without any experimental assistance, which is crucial for theoretical calculations. Our current work represents a refinement of HFR calculations guided by experimental reference data, thus possessing limited predictive power. To achieve full predictive capabilities, more sophisticated machine learning models with enhanced learning and generalization abilities, such as artificial neural networks [33], would be required. This aspect represents an important direction for future investigation.

## 4. Conclusions

We constructed a comprehensive, machine learning-assisted general workflow for atomic structure calculations. Within this framework, we developed and applied four machine learning methods combined with HFR theory for atomic structure calculations: RAEN, XGB-R, XGB-B, and XGB-C. These methods were used to calculate 56 energy levels of the Yb I, including eight even-parity configurations and six odd-parity configurations of the 4*f*^14^ closed shell. Under optimal conditions, the best-performing model demonstrates excellent predictive capabilities. When compared to NIST data, the model achieves an average absolute error of less than 50 cm^−1^ for even-parity configurations and less than 100 cm^−1^ for odd-parity configurations. Compared to HFR ab initio calculations, our method demonstrates significantly improved accuracy, while the lower *R*-value indicates enhanced computational stability. To achieve comparable accuracy levels, traditional ab initio methods would require more sophisticated theoretical models incorporating various correlation effects through complex perturbation terms. This approach would necessitate the use of more elaborate atomic structure calculation programs, substantially increasing computational resource requirements. Through our data-driven approach, we successfully enhanced calculation accuracy while maintaining the computational efficiency inherent to the Cowan code framework.

We analyzed the conditions where each method is most applicable. As additive models, XGB-like methods are highly accurate and generalizable for a large number of energy level calculations across clusters of energy levels, and can be used to determine the *steps* of changes in energy level structure, but the energy level data used for training fits need to be carefully selected to avoid overfitting. Our sample selection prioritizes energy level cluster boundary states to capture the overall characteristics of clusters, incorporates inter-configuration transition states to reflect inter-configuration interactions, and implements uniform sampling within clusters to select representative states. All selected energy levels are required to maintain high purity, where the leading configuration accounts for more than 80%. For closely spaced energy levels within a cluster where the number of calculations is relatively small, linear models such as RAEN can be employed. Additionally, these models allow for the optimization of HFR theory by updating the Slater parameters. Consequently, different methods of calculation can be used selectively for different atomic system characteristics and the availability of experimental energy level data. Furthermore, the flexible and extensible computational workflow enables the potential integration of a wider range of machine learning algorithms to assist in atomic structure calculations.

## Figures and Tables

**Table 1:** Yb I even-parity excited state energy levels (in unit cm^−1^) from the experiment (NIST), ab initio calculations, and fitting calculated of RAEN, XGB-R, XGB-B, and XGB-C, where ∆E (in unit cm^−1^) and R represent the absolute errors and root mean square errors between experimental and theoretical energies, respectively.

Config.	Term	*J*	Exp. [3]	Ab Initio	RAEN		XGB-R		XGB-B		XGB-C		Other Work
					Level	∆E	Level	∆E	Level	∆E	Level	∆E	
*4f^14^6s^2^*	^1^S	0	0.0	0.0	0.0	0.0	0.0	0.0	0.0	0.0	0.0	0.0	
*4f^14^5d6s*	^3^D	1	24,489.1	22,944.7	24,492.7	3.6	24,435.7	53.4	0.0	0.0	24,132.8	356.3	25,108 ^a^
	^3^D	2	24,751.9	25,366.7	24,757.7	5.8	24,765.2	13.3	24,364.8	124.3	24,893.1	141.2	25,368 ^a^
	^3^D	3	25,270.9	25,983.5	25,339.4	68.4	25,324.6	53.7	24,838.6	86.7	25,438.4	167.4	25,891 ^a^
	^1^D	2	27,677.6	26,630.8	27,676.0	1.6	27,661.4	16.2	25,352.4	81.5	27,436.3	241.3	28,353 ^a^
*4f^14^6s7s*	^3^S	1	32,694.7	31,957.0	32,697.4	2.7	32,700.5	5.8	27,626.3	51.3	32,524.2	170.5	33,092 ^a^
	^1^S	0	34,350.6	32,225.7	33,773.0	577.6	34,324.3	26.3	32,689.8	4.9	33,853.5	497.1	34,755 ^a^
*4f^14^6s6d*	^3^D	1	39,808.7	39,365.8	39,810.6	1.9	39,817.1	8.4	34,269.0	81.6	39,707.0	101.7	
	^3^D	2	39,838.0	39,387.1	40,091.6	253.6	39,866.6	28.6	39,858.3	49.6	39,734.5	103.5	
	^3^D	3	39,966.1	39,571.4	39,967.8	1.7	39,956.3	9.8	39,951.0	113.0	39,875.4	90.7	
	^1^D	2	40,061.5	39,608.5	40,397.6	336.1	40,058.3	3.2	39,919.7	46.4	39,956.6	104.9	
*4f^14^6s8s*	^3^S	1	41,615.0	40,730.6	41,616.3	1.3	41,612.4	2.6	40,068.3	6.8	41,410.7	204.3	
	^1^S	0	41,939.9	40,773.8	41,791.3	148.6	41,903.0	36.9	41,572.8	42.2	41,665.6	274.3	
*4f^14^6p^2^*	^3^P	0	42,436.9	43,448.4	44,134.3	1697.3	42,491.4	54.5	42,557.9	121.0	42,671.4	234.5	
	^3^P	1	43,805.4	44,199.7	43,882.8	77.4	43,820.2	14.8	43,900.4	95.0	43,897.2	91.8	
*4f^14^6s7d*	^3^D	1	44,311.4	43,384.2	44,303.4	8.0	44,302.7	8.7	44,259.9	51.5	44,098.0	213.4	
	^3^D	2	44,313.1	43,391.2	44,261.8	51.3	44,303.5	9.6	44,255.5	57.6	44,100.8	212.3	
	^3^D	3	44,357.6	43,459.1	44,092.7	264.9	44,360.8	3.2	44,347.7	9.9	44,150.6	207.0	
	^1^D	2	44,380.8	43,474.3	44,670.6	289.8	44,380.8	0.0	44,410.0	29.2	44,171.6	209.2	
*4f^14^6p^2^*	^3^P	2	44,760.4	45,030.0	44,770.0	9.6	44,360.8	3.2	44,869.5	109.1	44,823.3	62.9	
*4f^14^6s9s*	^3^S	1	45,121.3	44,097.2	44,775.4	345.9	45,115.8	5.5	40,068.3	6.8	41,410.7	204.3	
	^1^S	0		44,123.7	45,487.9		45,353.6		41,572.8	42.2	41,665.6	274.3	
*4f^14^6s8d*	^3^D	1	46,445.0	45,388.8	46,449.7	4.7	46,448.0	3.0	45,124.6	3.3	44,885.7	235.6	
	^3^D	2	46,467.7	45,387.6	46,458.4	9.3	46,441.4	26.3	45,259.0		45,075.0		
	^3^D	3	46,480.7	45,425.9	46,195.7	285.0	46,468.4	12.3	46,423.2	21.8	46,201.4	243.6	
	^1^D	2		45,424.8	46,727.1		47,551.1		46,382.0	85.7	46,218.0	249.7	46,405.45 ^b^
*4f^14^6p^2^*	^1^D	2	47,821.8	48,840.9	47,700.8	121.0	47,862.5	40.7	46,438.5	42.2	46,237.8	242.9	
	^1^S	0	45,121.3	49,525.3	50,963.7		50,631.6		47,492.1		47,078.8		
*R* *				966.1	439.2		24.7		75.0		247.8		

* *R* is the root mean square error (RMSE), and ^a^ Ref. [30], ^b^ Ref. [31].

**Table 2:** Small-sample even-parity excited state energy levels of Yb I calculated using Cowan fit, RAEN, XGB-R, XGB-B, and XGB-C methods, where ∆E (in unit cm^−1^) and R represent the absolute errors and root mean square errors between experimental and theoretical energies, respectively.

Config.	Term	*J*	Exp. [3]	Ab Initio	Cowan Fit	Other Work	RAEN		XGB-R		XGB-B		XGB-C	
							Level	∆E	Level	∆E	Level	∆E	Level	∆E
*4f^14^6p^2^*	^3^P	0	42,436.9	43,448.4	43,443.7		42,708.5	271.6	42,436.9	0.0	42,443.9	7.0	42,671.6	234.7
	^3^P	1	43,805.4	44,199.7	44,199.7		43,960.9	155.5	43,805.4	0.0	43,809.5	4.1	43,897.7	92.3
*4f^14^6s7d*	^3^D	1	44,311.4	43,384.2	43,340.4		44,293.9	17.5	42,366.3	1945.0	42,375.7	1935.7	43,463.2	848.2
	^3^D	2	44,313.1	43,391.2	43,373.7	44,314.1 ^c^	44,294.2	18.9	44,313.1	0.0	44,301.2	11.9	44,101.3	211.8
	^3^D	3	44,357.6	43,459.1	43,411.9	44,345.3 ^c^	44,343.5	14.1	44,357.6	0.0	44,353.3	4.3	44,150.5	207.1
	^1^D	2	44,380.8	43,474.3	43,152.7	44,401.1 ^c^	44,366.3	14.5	44,380.8	0.0	44,378.8	2.0	44,172.8	208.0
*4f^14^6p^2^*	^3^P	2	44,760.4	45,030.0	44,466		44,879.8	119.4	46,034.7	1274.3	46,027.7	1267.3	44,836.1	75.7
*4f^14^6s9s*	^3^S	1	45,121.3	44,097.2	44,070.6		45,074.2	47.1	45,121.3	0.0	45,115.5	5.8	44,886.0	235.3
	^1^S	0		44,123.7	44,092.7		45,909.5		45,361.0		45,346.9		45,076.2	
*4f^14^6s8d*	^3^D	1	46,445.0	45,388.8	45,376.4		46,364.7	80.3	46,445.0	0.0	46,433.4	11.6	46,202.1	242.9
	^3^D	2	46,467.7	45,387.6	45,392.7		46,377.7	90.0	44,984.3	1483.4	44,990.4	1477.3	44,840.9	1626.8
	^3^D	3	46,480.7	45,425.9	45,412.6		46,398.0	82.7	46,480.7	0.0	46,470.5	10.2	46,238.2	242.5
	^1^D	2		45,424.8	45,292.8		47,550.9		47,574.0		47,552.6		47,062.2	
*4f^14^6p^2^*	^1^D	2	47,821.8	48,840.9	46,097.7		47,961.3	139.5	47,822.8	1.0	47,832.4	10.6	48,059.5	237.6
*R* *					1040.8		114.1		737.6		734.3		604.4	

* *R* is the root mean square error (RMSE), and ^c^ Ref. [32].

**Table 3:** Partial Slater parameters (in unit cm^−1^) from Cowan fit and RAEN fitting calculations of small-sample even-parity excited state energy levels of Yb I.

Linear Model	Cowan Fit	RAEN
$E_{av}$(4*f*^14^6*s*7*d*)	43,385.3	42,878.3
$\zeta_{f}$	28.6	28.7
${G^{2}}_{sd}$	28.6	62.6
$E_{av}$(4*f*^14^6*p*^2^)	44,875.8	45,269.1
${F^{2}}_{pp}$	1752	1395.8
$\zeta_{d}$	931.8	241.4
$E_{av}$(4*f*^14^6*s*8*d*)	45,399.2	45,770.5
$\zeta_{f}$	14.5	13.5
${G^{2}}_{sd}$	13.7	16.9
$E_{av}$(4*f*^14^6*s*9*s*)	44,076.9	44,424.7

**Table 4:** Yb I odd-parity excited state fitting calculated energy levels (in unit cm^−1^) using RAEN, XGB-R, XGB-B, and XGB-C methods, where ∆E (in unit cm^−1^) and R represent the absolute errors and root mean square errors between experimental and theoretical energies, respectively.

Config.	Term	*J*	Exp. [3]	Ab Initio	RAEN		XGB-R		XGB-B		XGB-C		Other Work
					Level	∆E	Level	∆E	Level	∆E	Level	∆E	
*4f^14^6s6p*	^3^P	1	17,992.0	17,891.7	17,808.4	183.6	17,979.1	12.9	17,913.1	78.9	17,968.9	23.1	18,450.0 ^a^
	^3^P	2	19,710.4	18,854.4	19,403.7	306.7	19,621.2	89.2	19,610.2	100.2	19,512.4	198.0	20,251.0 ^a^
	^1^P	1	25,068.2	29,170.4	26,212.4	1144.2	25,167.1	98.9	25,182.8	114.6	26,015.2	947.0	25,967.0 ^a^
*4f^14^6s7p*	^3^P	0	38,090.7	39,090.3	38,305.8	215.1	38,135.3	44.6	38,199.4	108.7	38,322.0	231.3	
	^3^P	1	38,174.2	39,183.5	38,173.9	0.3	38,200.6	26.4	38,232.3	58.1	38,407.2	233.0	
	^3^P	2	38,552.0	39,400.8	38,804.1	252.1	38,558.0	6.0	38,573.6	21.6	38,747.8	195.8	
	^1^P	1	40,564.0	40,914.4	40,561.9	2.1	40,540.9	23.1	40,517.7	46.4	40,644.8	80.8	
*4f^14^6s5f*	^3^F	3		44,148.2	43,259.5		43,273.0		43,286.2		43,466.8		43,297.51 ^c^
	^3^F	4		44,148.5	41,711.7		43,379.1		43,367.3		43,560.9		
	^3^F	2	43,433.9	44,148.0	43,853.2	419.3	43,452.1	18.2	43,509.9	76.0	43,598.7	164.8	
	^1^F	3		44,209.3	43,519.2		43,460.8		43,441.2		43,673.4		43,254.8 ^c^
*4f^14^6s8p*	^3^P	0	43,614.3	44,595.7	43,556.8	57.5	43,628.3	14.0	43,635.2	20.9	43,840.4	226.1	
	^3^P	1	43,659.4	44,632.7	43,597.0	62.4	43,670.3	10.9	43,708.9	49.5	43,884.1	224.7	
	^3^P	2	43,805.7	44,719.5	43,879.0	73.3	43,886.7	81.0	43,908.8	103.1	44,017.0	211.3	
	^1^P	1	44,017.6	45,308.4	43,918.4	99.2	44,098.7	81.1	44,131.7	114.1	44,315.8	298.2	
*4f^14^6s6f*	^3^F	2	45,956.3	46,777.3	45,821.5	134.8	45,942.4	13.9	45,957.6	1.3	46,145.1	188.8	
	^3^F	3		46,777.5	46,421.1		46,018.1		45,991.2		46,219.9		
*4f^14^6s9p*	^3^P	1	46,078.9	47,040.5	46,317.6	238.7	46,185.5	106.6	46,203.7	124.8	46,301.8	222.9	
	^3^P	0	46,082.2	47,021.3	46,082.6	0.4	46,134.3	52.1	46,180.3	98.1	46,299.0	216.8	
*4f^14^6s6f*	^3^F	4		46,777.6	45,858.3		46,031.8		46,018.6		46,271.9		
	^3^F	3		46,818.4	46,042.6		46,051.5		46,009.9		46,319.7		
*4f^14^6s9p*	^3^P	2	46,184.2	47,084.7	46,184.3	0.1	46,211.5	27.3	46,243.7	59.5	46,391.9	207.7	
	^1^P	1	46,370.3	47,435.3	46,842.4	472.1	46,545.1	174.8	46,541.8	171.5	46,617.0	246.7	
*4f^14^6s6f*	^3^F	2	47,326.7	48,186.2	47,326.7	0.1	47,313.0	13.7	47,345.2	18.5	47,525.3	198.7	
	^3^F	3		48,186.3	47,525.1		47,419.4		47,377.8		47,637.2		
	^3^F	4		48,186.4	47,764.0		47,776.1		47,762.6		47,938.9		
	^1^F	3		48,212.8	48,062.6		48,169.5		48,155.7		48,247.1		
*R* *				1302.1	338.8		67.2		88.8		297.2		

* *R* is the root mean square error (RMSE), and ^a^ Ref. [30], ^c^ Ref. [32].

## Data Availability

Datasets generated during the current study are available from the corresponding authors on reasonable request.
